# 

**Published:** 1888-12-22

**Authors:** 


### CONTENTS

**Givers and Giving** - 177

**The Student's Column.**—The University of London - 178

**The Doctor's Pulpit.**—Present-Day Problems.—Cures for "Social Parasites" - 179

**Turning Over New Leaves.**—"The Universal Review."—Plays for Children - 180

**Something About "Germs." The Views of Professor Charteris of the Glasgow University** - 181

**"First Aid to the Injured"** - 182

**Notes and News** - 184

**Voyages in Vacation.**—XIII. Royal Carriages—Railways—Moscow - 183

**Everybody's Page** - 18[illegible]

**Annotations.**—A New Opening for Nurses.—London Boy Ruffianism—Funeral Wreaths - 187

**False Impressions.** By CAROLINE FOTHERGILL, author of "An Enthusiast," "A Voice in the Wilderness." Chap. XII. 188

**Hospital Extravagance and Expenditure** - 189

**Scraps and Gleanings** - 192

**Amusements and Relaxation** - 192

**EXTRA SUPPLEMENT.—THE NURSING MIRROR.**

**En Passant.**—Concert—Decorations.—Nurses and Advertisements.—Conversaziones—Suicide of a Nurse.—Hurrah for the Pension Fund!—District Nurses.—The British Nurses' Association - xlv
**Town Nursing.**—A Scheme for Imitation - xlvi
**Original Articles.**—The Shut In Society - xlvi
**Presentation.—Notice** - xlvii
**Wages and Washing.**—IV—Concluded - xlvii
**Everybody's Opinion.**—An Appeal for Christmas Help, etc. xlviii
**Jottings—Vacancies—Notes and Queries** - xlviii

